# Integrated plasmon-enhanced Raman scattering (*i*PERS) spectroscopy

**DOI:** 10.1038/s41598-017-15111-3

**Published:** 2017-11-07

**Authors:** Hailong Wang, Haibo Li, Shuping Xu, Bing Zhao, Weiqing Xu

**Affiliations:** 1 State Key Laboratory of Supramolecular Structure and Materials, Institute of Theoretical Chemistry, Jilin University, Changchun, 130012 People’s Republic of China; 2 State Key Laboratory of Supramolecular Structure and Materials, College of Chemistry, Jilin University, Changchun, 130012 People’s Republic of China

## Abstract

A new strategy named integrated plasmon-enhanced Raman scattering (*i*PERS) spectroscopy that features a configuration of evanescent field excitation and inverted collection is presented, which well unites the local field enhancement and far field emission, couples localized and propagating surface plasmons, integrates the SERS substrates and Raman spectrometers via a self-designed aplanatic solid immersion lens. A metallic nanoparticle-on-a film (NOF) system was adopted in this configuration because it improves the amplification of the incidence light field in near field by 10 orders of magnitude due to the simultaneous excitation of quadrupolar and dipolar resonance modes. This iPERS allows for higher excitation efficiency to probed molecules and full collection of the directional-radiation Raman scattering signal in an inverted way, which exhibits a practical possibility to monitor plasmonic photocatalytic reactions in nanoscale and a bright future on interfacial reaction studies.

## Introduction

Surface-enhanced Raman spectroscopy (SERS) is a unique optical phenomenon that can improve the Raman signal of molecules millions times and has gained more and more attentions within the last forty years^1–4^. Electromagnetic (EM) enhancement is one of two widely accepted SERS enhancement mechanisms which amplifies the Raman signal of the probed molecules adjacent to the local electric field due to the surface plasmon resonance (SPR) effect^2–4^. Thus, stronger local electric field amplification is always pursued by researchers and almost becomes a golden rule for evaluating every SERS substrate. However, other than near field coupling, a method to collect radiative SERS signals in far field is also vital in order to achieve high detection sensitivity. This is often overlooked in many SERS studies because the Raman setup and SERS substrates were typically considered separately, making them mismatched, causing the far field collection to be ignored.

Resonating the surface plasmon wave with the incident light vector plays a decisive role in all plasmon-based spectroscopies and sensing, e.g. metal-enhanced fluorescence and SERS. In various plasmonic materials and structures, resonance is embodied in different forms, for instance, in the prism-type propagating surface plasmon resonance configurations, resonance can cause nearly 100% light trapping, which has been applied for the excitation of fluorophores^5–7^ and Raman photons^8–12^. However, signal collection for radiators are usually ignored. It should be noted that these structures feature directional plasmon radiation^13–16^. If a commonly used Raman spectroscopic setup featuring a fixed backscattering or inverted configuration is used for collecting SERS signals from these SERS substrates, only a part of SERS signals is collected. To increase the signal collection ratio, both the signal excitation and collection methods must be considered.

Here, a unique way that improves the SERS excitation and emission is presented by developing an integrated plasmon-enhanced Raman scattering (*i*PERS) spectrometer using a specially designed aplanatic solid immersion lens (ASIL, NA ≈ 1.65). A metallic nanoparticle-on-a film (NOF) architecture was layered on the ASIL. Owing to the evanescent field excitation and inverted collection (i.e. attenuated total reflection configuration, ATR), the electrical field enhancement in near field and the directional collection of SERS in far field can be fulfilled simultaneously. The circular emission cone of the directional SERS signals in space can be fully collected *via* this ASIL, improving signal collection efficiency, which is extremely important for monolayer identification and surface analysis.

## Results and Discussion

### Photonics of *i*PERS

**Figure 1:**
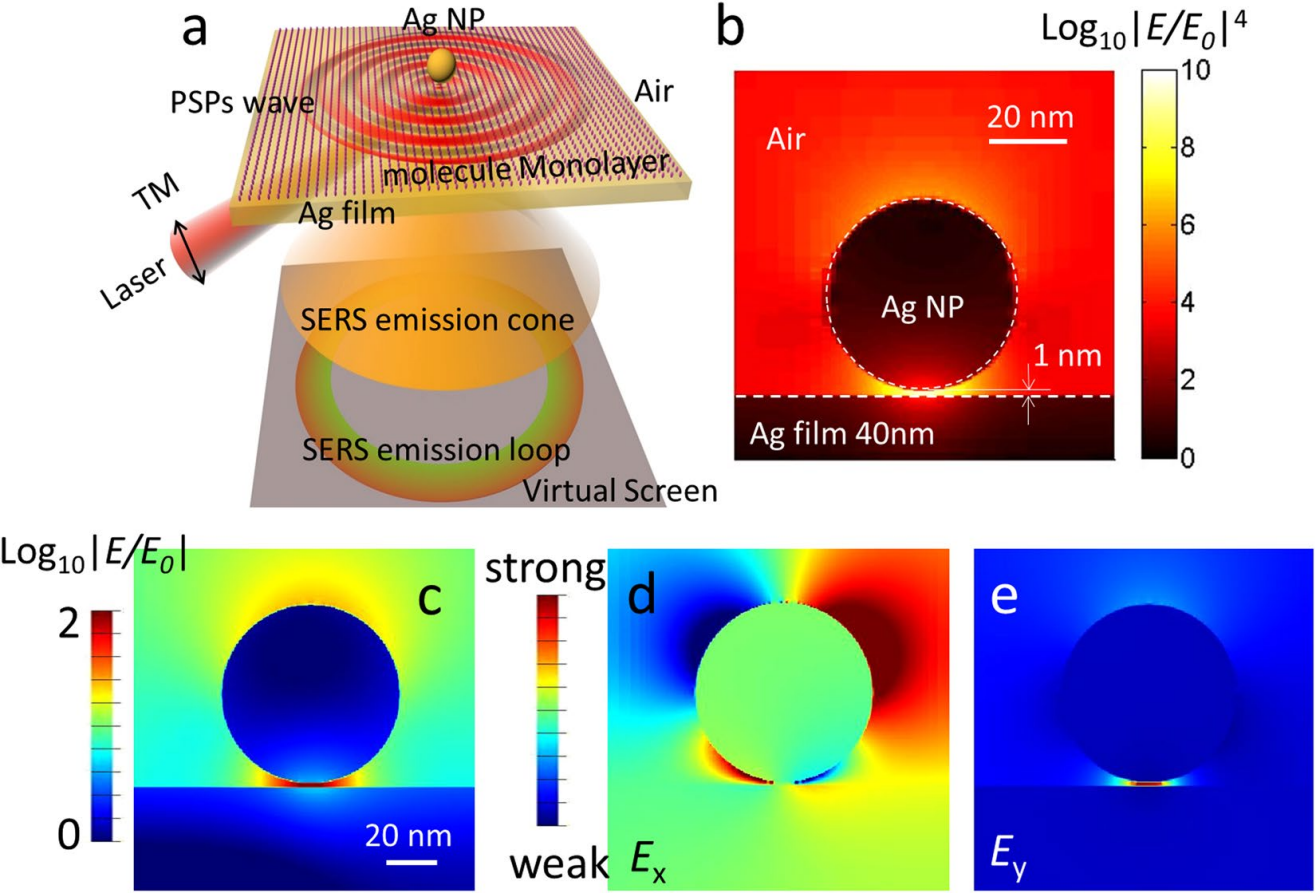
The excitation and emission of SERS in our designed *i*PERS configuration and the local electric field enhancement from NOF substrate. (**a**) Schematic diagram of the directional excitation and emission of the SERS of the monomolecular layer located in the gap of an Ag NP-on-a film system (NOF). (**b**) SERS enhancement distribution of the NOF in near field simulated by three-dimensional-finite-difference time-domain (3D-FDTD) software. The NOF used in this research is constructed by an Ag NP (diameter: 50 nm) over an Ag film (thickness: 40 nm). The gap between the Ag NP and film is dependent on the thickness of the monomolecular layer located on the surface of the Ag film. Here, we set the gap as 1.0 nm according to the frequently probed molecules in SERS detections. The excitation laser is 785 nm. (**c**) The electric field enhancement distribution of the NOF and its components in E_x_ (**d**) and E_y_ (**e**) simulated by 2D-FDTD software, showing the simultaneous excitation of quadrupole (**d**) and dipole (**e**) resonance modes.

The physical process in *i*PERS spectroscopy (shown in Fig. 1a) involves two steps: (1) the coupling the localized surface plasmons (LSPs) in near field, and (2) the SERS emission in far field. Firstly, the incident laser in *p* polarization (785 nm) couples the propagating surface plasmons (PSPs) on an Ag film in a narrow angle range, at which the transverse propagation vector of the incident laser matches with PSPs (namely the vector-match law^17^, see S1). When the PSPs encounter an Ag NP on an Ag film, the LSPs in the nanogap will be coupled. Secondly, the LSPs excite the Raman scattering of the probed molecules in the nanogap, and the scattering light couples the PSPs of the Ag film again. Next, the PSPs radiate in far field along a certain cone angle according to the vector-match law. Thus, an emission loop comes out and a cone pattern of the SERS emission can be obtained in far field^18–21^. In this design, the coupling of LSPs in near field and the SERS emission in far field are integrated by an elaborately designed ASIL.

The EM fields in near and far fields were firstly estimated by finite-difference time-domain (FDTD) simulation. An Ag NP (diameter: 50 nm) on an Ag film (thickness: 40 nm) configuration is excited by a 785-nm laser from the ASIL side. Under the resonance condition, a hot spot (shown as the red region) appears at the nanogap (1 nm) in this NOF configuration. The SERS enhancement distribution in Fig. 1b shows the enhancement factor of SERS (|E/E_0_|^4^) reaches 10 orders of magnitude in the hot spot region. Figure 1c displays the electric field distribution of the NOF and its components in E_x_ and E_y_ (Fig. 1d and e), respectively. It can be found that quadrupole (Fig. 1d) and dipole (Fig. 1e) resonance modes can be simultaneously excited, which can answer for why such high EM field can be reached in the gap range in this NOF configuration^22^.

**Figure 2:**
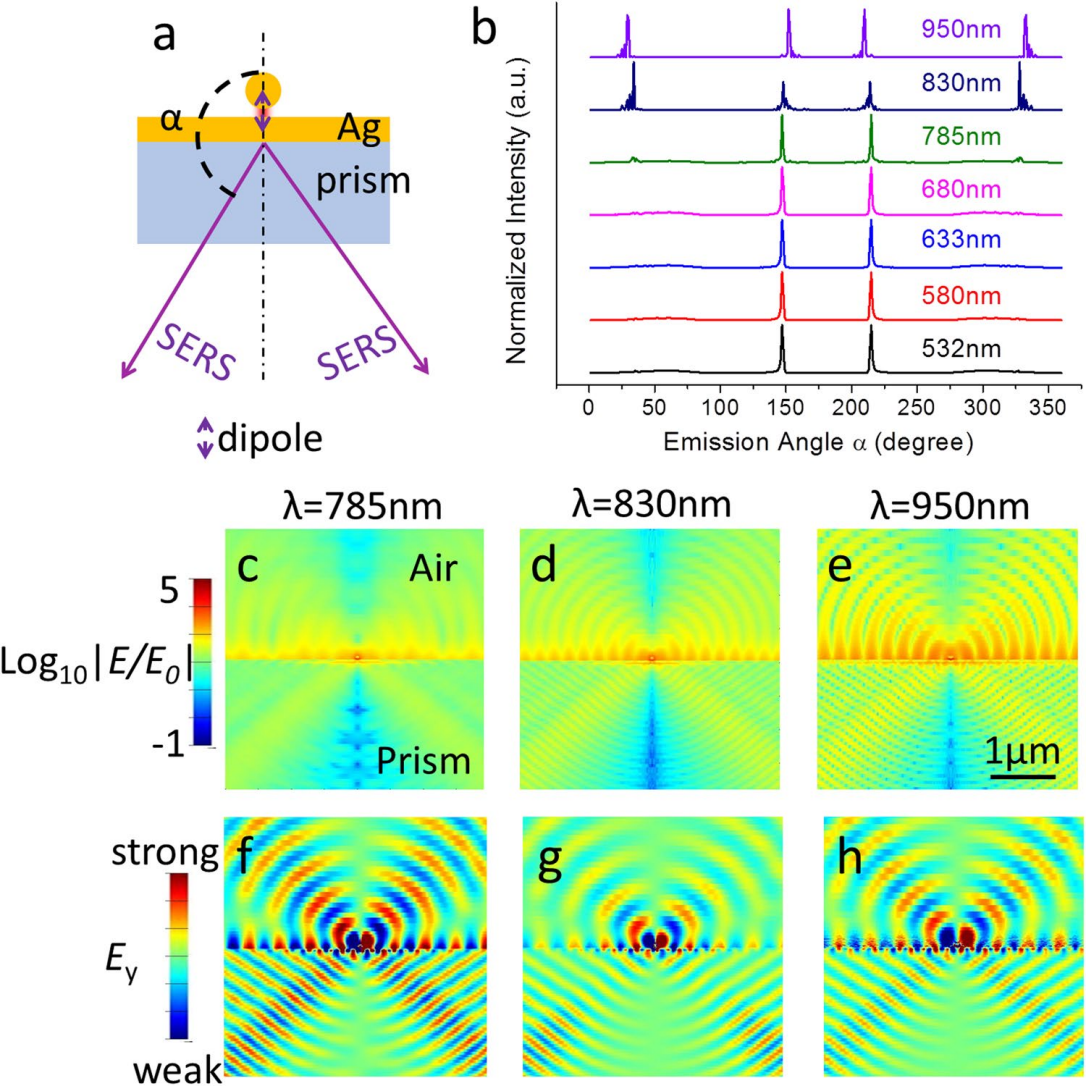
Directional emission patterns of different wavelength dipoles located in the gap of the NOF to mimic the radiation process of Raman photons (**a**) Schematic diagram of the model for 2D-FDTD simulation. A dipole is located in the nanogap (1.0 nm), and α is the emission angle of the radiation ray (marked as “SERS” in (**a**)). (**b**) Angle-dependent emission of different wavelength dipoles (532–950 nm) in far field. Most of photons radiate as a sharp cone angle in the prism side. (**c**–**e**) The far field electric field distributions of dipoles with different wavelengths. The directional emission of SERS is also clearly shown in (**f**–**h**), while the component of electric field is parallel to the Ag film.

The directional SERS emission in far field is the second kernel in this *i*PERS design. To mimic the radiation process of Raman photons, the probed molecule is represented as a dipole that is located in the gap and radiates light at the wavelengths from 532 to 950 nm (Fig. 2a). Fig. 2b shows the angle sweeps of SERS emission intensities of several Raman photons collected from far field. It can be found that the emission angles sharply focus into the narrow angle range and only part of the scattering signal appears in the air side, especially for the emitter with the wavelengths shorter than 830 nm^18,23^. The radiative patterns of the EM fields of 785, 830 and 950 nm radiators in the gap of the NOF are shown in Fig. 2c–e and the corresponding E_y_ are shown in Fig. 2f–h (The far field electric field distributions of dipoles with other wavelengths are shown in S2). It can be observed that strong radiative EM fields in air side are only localized at the air/prism interface, and they remarkably decay with distance. Therefore, in the air side, we hardly collect the signals of radiators in far field. In contrast, the prism side allows for weak decay in the EM field intensity, and narrower divergence angle is achieved, which ensures the directional emission of the SERS signal in the prism side and provides possibility for signal collection in high efficiency using the inverted way. This directional emission phenomenon has been proved to be originated from the emission of the PSPs of the Ag film on the prism in our previous study^16^. The directional emission patterns of different wavelength dipoles located over the Ag film are also provided in S3, in which stronger directional emission in prism side is observed due to the PSP coupling.

### Optics of iPERS

**Figure 3:**
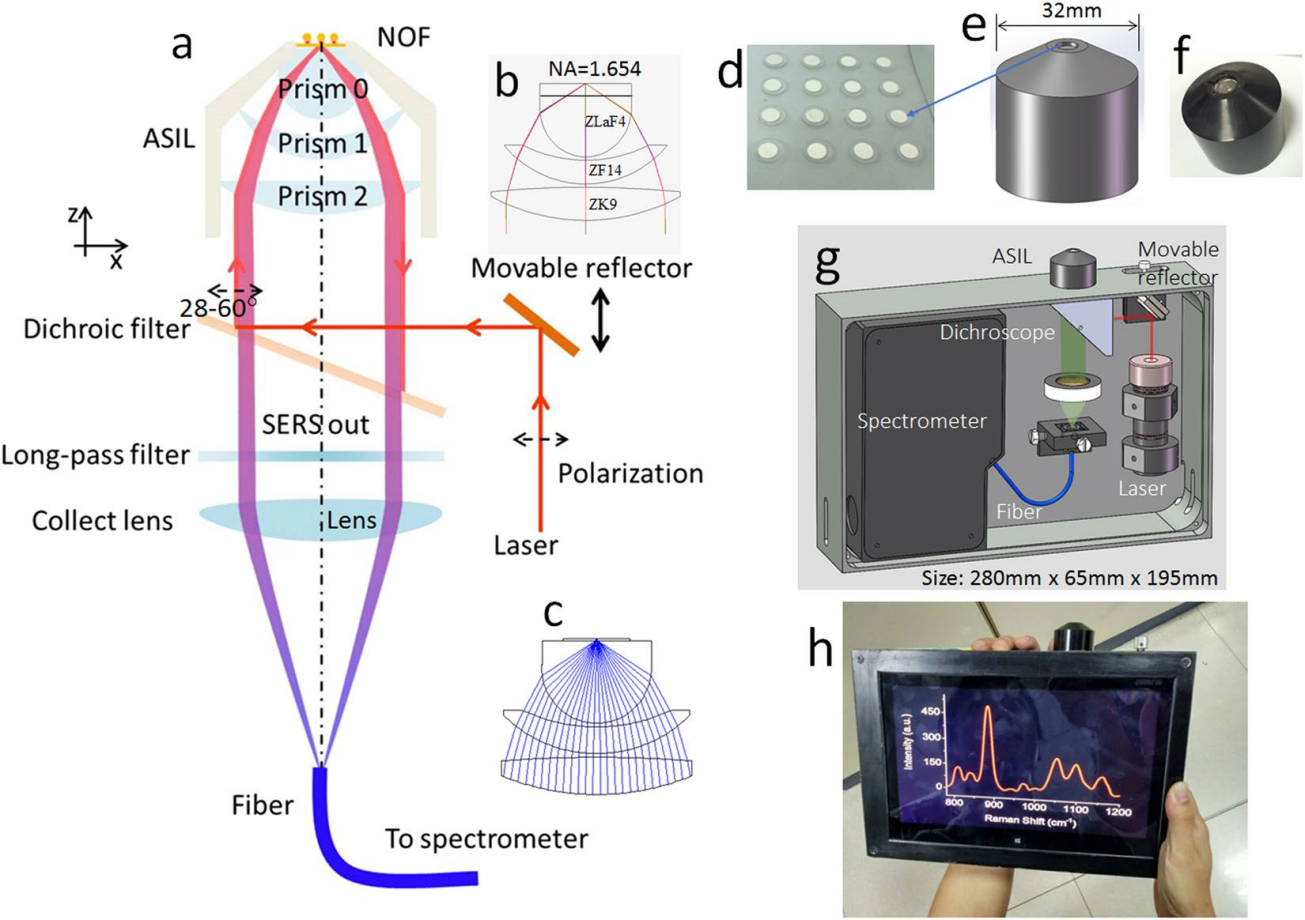
The optical and mechanical structure of the *i*PERS instrument. (**a**) The ASIL and the optical system designed for the *i*PERS instrument. (**b**) The optical configuration of the ASIL. (**c**) The light path in the ASIL. (**d**) Photo of the NOF substrates. (**e**) and (**f**) are the 3D mechanical model and the photo of the ASIL, respectively. The numerical aperture of ASIL is 1.654. The whole size of the *i*PERS setup is 280 mm × 65 mm × 195 mm. (**g**) and (**h**) are the 3D mechanical model and the photo of the *i*PERS instrument.

In order to satisfy the excitation angle and the collection of the directional SERS simultaneously, the inverted optical path was designed as shown in Fig. 3a. The dominating part of *i*PERS is an ASIL, which can expand the numerical aperture (NA) aplanaticly according to the theory of aplanatic lenses^24^. The laser spot for sample is 4 μm in diameter in theory due to the utilization of high refractive index prisms (see Part S5). With the assistance of the dichroic filter and the movable reflector, the incident laser goes into the ASIL and focuses on the upper surface of the metallic NOF structure (Fig. 3b). The resonance angle for Ag is about 32 degree (Fig. S6). Molecules excited in the gap directionally radiate Raman photons and recouples the PSPs. PSPs with different frequencies in prism side are transformed into a collimated beam and then collected by the Raman spectrometer with a long-pass filter and prism 3. The descriptions about the optical details are provided in experimental section and the verifying of this optical structure is described in Part S4. The system is controlled by the LabVIEW program (National Instruments Co.) on a tablet computer. This *i*PERS setup is in the size of 280 mm × 65 mm × 195 mm with the weight of 4.5 kg.

### Detection of monolayered molecules by iPERS

**Figure 4:**
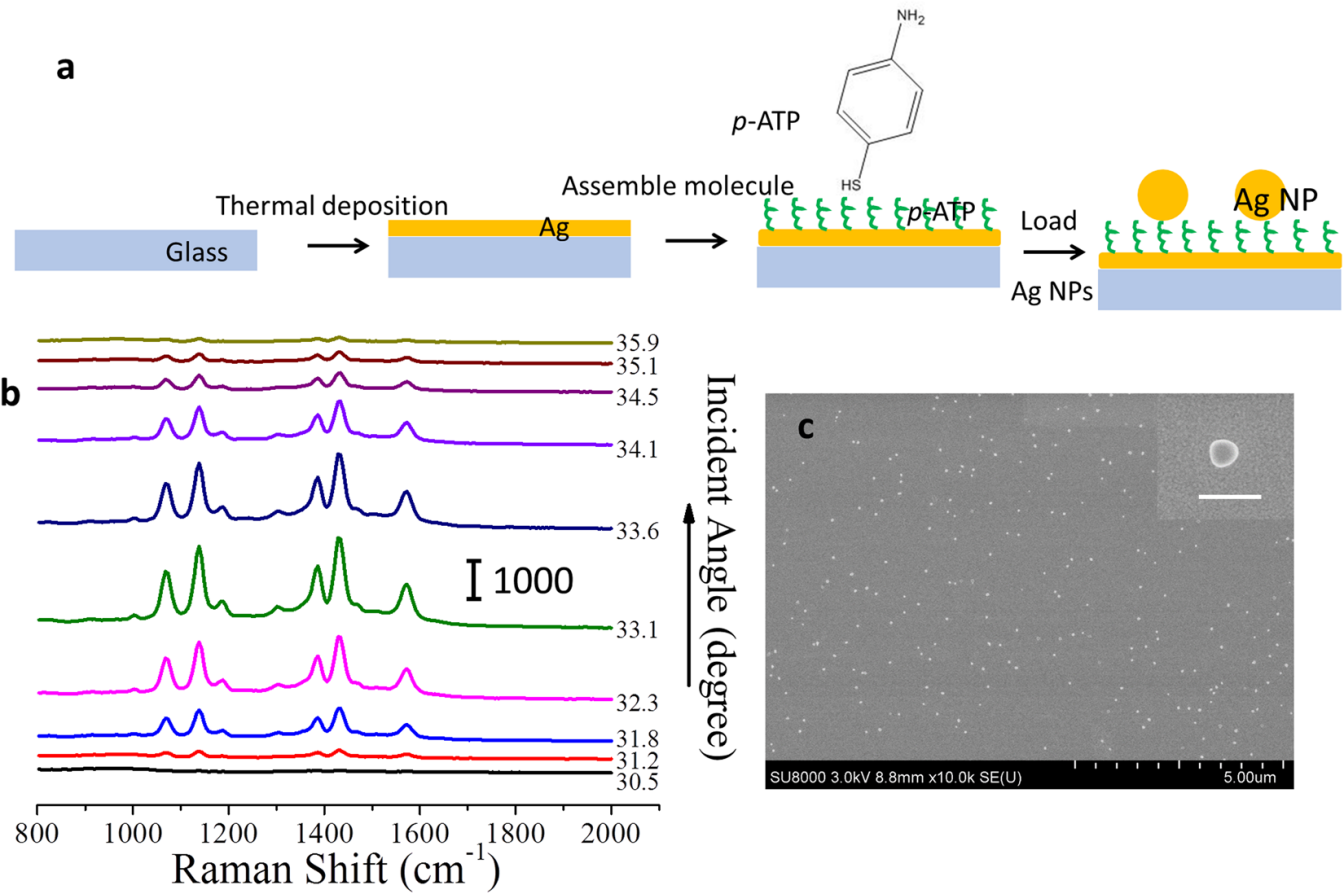
Fabrication process and the angle-resolved SERS spectra of NOF substrate. (**a**) Fabrication process of the NOF substrate. The Ag film is thermally deposited onto the surface of the glass substrate. Then the *p*-ATP monomolecular layer and the Ag NPs are assembled on the surface of the Ag film successively. (**b**) The SERS spectra of the *p*-ATP monomolecular layer detected at different incident angles. Laser power was 11.0 mW and the integrated time was 10 s. (**c**) The SEM image of the NOF structure fabricated in (**a**). The insert is the SEM of An Ag NP of the NOF substrate. Scale bar is 100 nm. The coverage of the NP on the NOF substrate is about 1.71/μm^2^ (0.76% in duty ratio).

**Figure 5:**
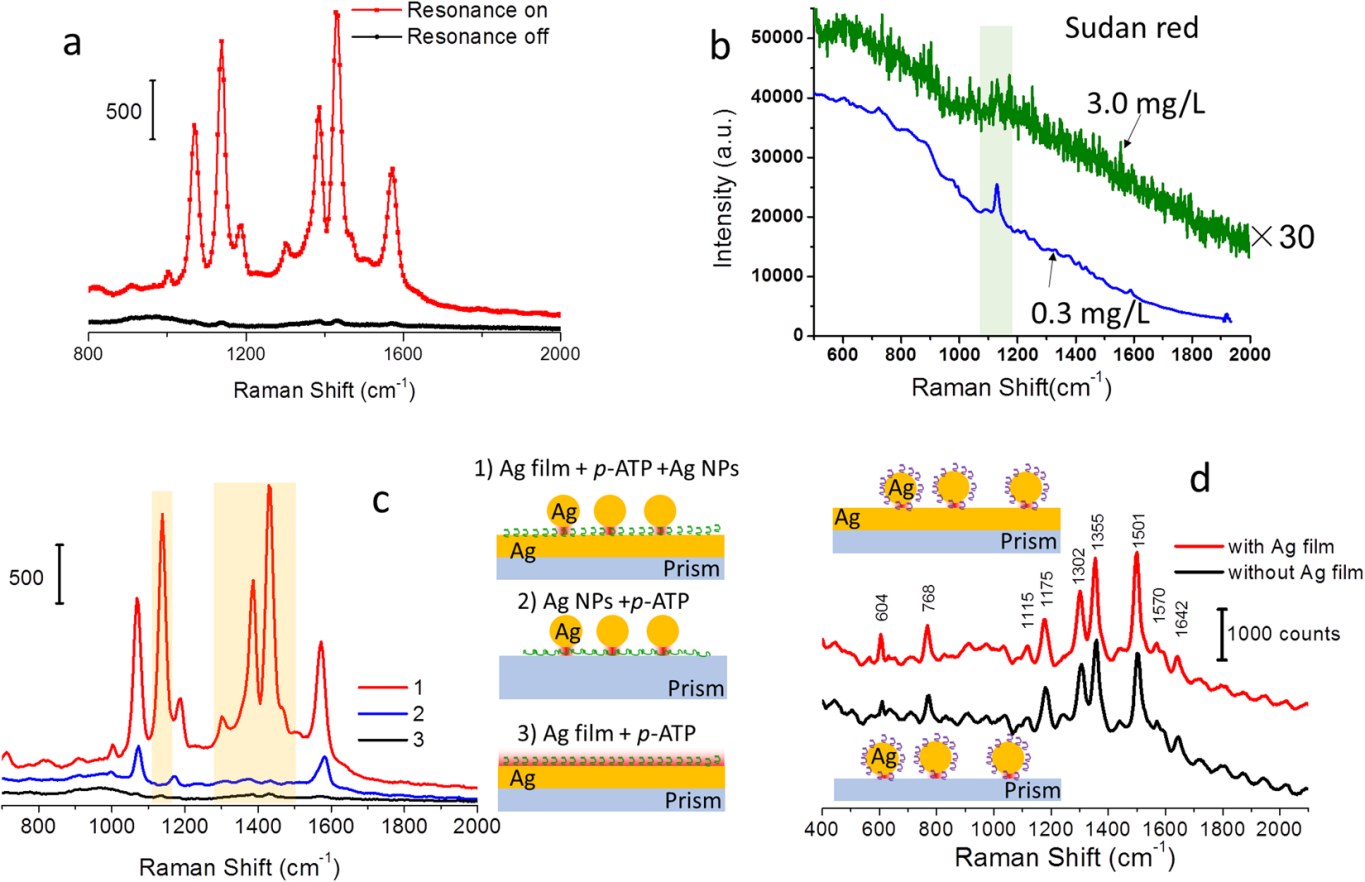
Performance assessment for the *i*PERS instrument. (**a**) SERS spectra of the monolayered *p*-ATP molecules on the NOF substrate with and without the SPR occurring. (**b**) Comparison between a commerical Raman instrument (B&Wtek portable optical fiber Raman spectrometer, B&Wtek 785) and the *i*PERS instrument. The Raman signal (top curve) is 30 times amplified. The integration time was 2 s. (**c**) SERS analysis of the monolayered *p*-ATP molecules assambled on different substrates, detected by the iPERS instrument. Three models of the SERS substrates: (1) the Ag film+*p*-ATP+Ag NPs model on NOF substrate, (2) the Ag NPs+*p*-ATP and (3) the Ag film+*p*-ATP model, respectively. The red regions in 1)–3) mark the “hot” areas contributing to SERS enhancement. The laser power was 11.0 mW and the integrated time was 10 s. Peaks highlighted in light brown are asigned to the DMAB molecule and the other peaks belong to *p*-ATP in (**c**). (**d**) The SERS spectra of rhodamine 6 G (R6G) decorated Ag NPs measured under the ATR excitation mode (bottom) using the ASIL and *i*PERS configuration (top).

We employed this *i*PERS setup to measure the monolayered molecules to test its sensitivity. The NOF substrate was adopted in *i*PERS, composed of 50 nm Ag NPs over a 40 nm thick Ag film. As shown in Fig. 4a, a monolayer of *para*-aminothiophenol (*p*-ATP) was assembled on the Ag film by bonding Ag with the thiol group of *p*-ATP molecule, and the Ag NP and the *p*-ATP layer were linked *via* the bond between Ag and the amino group of *p*-ATP molecule. Figure 4c is the SEM image of the NOF substrate. We can observe that the separation distance between adjacent Ag NPs is large enough to avoid the plasmonic coupling between them. The density of the NPs is about 1.71/µm^2^ (duty ratio is 0.76%). The angle-resolved SERS spectra of the *p*-ATP monolayer detected by the *i*PERS were recorded and shown in Fig. 4b, in which the SERS intensities obviously change when the incident angle sweep, which is consistent with the simulation results that this ATR excitation mode allows for the directional emission of SERS. At the resonance angle, the SERS intensity of *p*-ATP molecule is strongest^22,23^. It should be noted that these peaks are expected to have a maximum intensity at different resonance angles due to different wavelengths as Fig. 2b (around 32°). However, in this experiment, they are too close to split. In Fig. 5a, the comparison of SERS spectra under resonance and non-resonance clearly proves a significant SERS enhancement at the SPR condition. If the coverage of Ag NPs is considered, the SERS signal enhancement might be optimized to be a higher value correspondingly (~100 times improvement at most if building a close-packing Ag NP array with a highest coverage of ~74%). We also compared the performance of *i*PERS with a commercial instrument (B&Wtek-785) by measuring Sudan red. As shown in Fig. 5b, the sensitivity of *i*PERS is 300 times larger than that of commercial instrument. To further exhibit the SERS behaviors of the NOF structure, three different plasmonic configurations are compared (1–3 Fig. 5c) with *p*-ATP as the probe. We can clearly find that the SERS spectrum from the NOF substrate has more peaks and the intensity is much stronger than that on the Ag film. Its intensity is 6 times stronger than that with the dispersed Ag NPs. The SERS peaks in Fig. 5b are mainly assigned to vibration modes of 4,4′-dimercaptoazobenzene (DMAB, a dimer of *p*-ATP produced by a plasmon-assisted photocatalysis reaction)^25–27^. In addition, the SERS spectra of rhodamine 6 G (R6G) decorated Ag NPs measured under the ATR excitation mode (bottom curve in Fig. 5d) using the ASIL and the *i*PERS configuration (top curve in Fig. 5d) were compared. It can be found that the NOF substrate has almost same SERS enhancement effect with the ATR-LSPs coupling configuration that can provide an enhancement field comparable to hot spot in our previous study^28^. These data indicate this configuration displays enough sensitivity to single layered molecules and can be used for monitoring interfacial reactions.

## Conclusions

In summary, a proof-of-concept integrated spectrometer (*i*PERS) was developed. For the compatibility between the SERS substrate and the detection instrument, the ASIL was specially designed for the directional excitation and emission of the SERS signal, as well as the full collection of the radiation cone of the Raman scattering in an inverted way. NOF was selected as the SERS substrate because of its signal enhancement capability based on the coupling of multi resonance modes and the directional emission of Raman scattering. The SERS signal of the *p*-ATP from the gap on the NOF substrate confirms the feasibility to detect monolayered molecules. This *i*PERS paves the way for the further development of SERS applications, especially for monitoring chemical and biochemical reactions in nanoscale.

## Experimental Section

### EM Simulations

FDTD methods (FDTD solution software, Lumerical, Canada) were used to calculate EM distributions in near field and far field and the directional emission of different wavelength dipoles located in the nanogap. 3D simulated regions were adopted when the localized EM enhancement on the NOF substrate was evaluated as shown in Fig. 1b. The refractive index of the glass substrate is set to 1.92 according to prism 0 of the ASIL as shown in Fig. 3a. An Ag NOF substrate is utilized. The NP diameter and thickness of the Ag film are 50 nm and 40 nm, respectively, defined by the fabrication process. 3D-FDTD simulation region was set as 1 μm × 1 μm × 3 μm along the X, Y, and Z axes, respectively. To simplify the simulation, boundary conditions in the X and Y directions are periodic and perfect match layer (PML) absorbing boundary condition is used along the Z axis. The mesh sizes of 0.1 and 2 nm are set in the gap and Ag NP region.

In order to reveal the EM resonance of the NOF substrate, simplified simulations in 2D are carried out with FDTD solution as shown in Fig. 1c–e. The boundary conditions are PML and periodic in the X and Z axis. Simulation regions are set to 1 μm × 3 μm along the X and Z axes. The mesh sizes of 0.1 and 1 nm are set in the gap and NP region.

The directional emission of SERS in far field is simulated with FDTD solution while the dipoles with different wavelengths are set in the 1.0 nm-nanogap. The resonance direction of the dipole is vertical to the Ag film and the EM scattering in far field is collected. To simplify the simulation, 2D FDTD is adopted and symmetric boundary conditions and PML are set in X and Z axis. The simulation regions are set to 30 μm × 3 μm in the X and Z axes. The mesh sizes of 0.1 and 1 nm are set in the gap and NP region.

### Sample Fabrication

An Ag film with the thickness of 40 nm was thermally deposited on the glass substrate (n = 1.92), which was then immersed into an ethanol solution of para-aminothiophenol (*p*-ATP, 1.0 × 10^−4^ M) for 3 h. By rinsing with the ethanol and drying with nitrogen, the extra *p*-ATP molecules were cleaned off and a monomolecular layer of *p*-ATP was formed on the Ag film *via* chemical adsorption^23^. Then the Ag film with *p*-ATP was immersed in an Ag colloidal gel (diameter: 50 nm; Nanjing XFNANO Materials Tech Co., Ltd) for 0.5 h, rinsed with ethanol and dried with nitrogen. Ag NPs were assembled on the surface of Ag film *via* the chemical adsorption.

### Optic Details of *i*PERS

Considering the angle requirement of the incident laser to excite the SERS through the ASIL, a movable reflector is introduced into the optical system of *i*PERS, as shown in Fig. 3a. The incident laser with the polarization along X axis is reflected by the movable reflector and the dichroic filter into the ASIL. By tuning the movable reflector in Z axis, the incident angle into the NOF surface will be modulated to match the surface plasmon resonance (SPR). Then the directional emission of SERS is filtered by the dichroic filter and the long-pass filter. Finally, the filtered SERS signal is focused into a fiber and guided into the detector.

The excitation source is the linear polarized laser with the wavelength of 785 nm (Single Frequency Collimated Laser Module, RO-785-PLR-80-1, Ondax, Inc, USA). The polarization direction is parallel to the incident plane of the laser into the surface of the SERS slide. The movable reflector (Broadband Dielectric Mirrors, GCC-101124, Daheng Optics, Inc, China) is fixed on a 1-axis moving stage and changes the incident angle when the stage is moving in Z axis. The dichroic filter (LPD01-785R, Semrock, Inc, USA) is installed on a beam splitter mirror supporter which is designed to fit the compact volume of the *i*PERS instrument. The structure parameters of the lenses used in the ASIL are optimized by the optical design software (Zemax-EE, Zemax LLC, USA) in consideration of the high NA and low aberration. The glass materials are ZLaF4 (n = 1.92, CDGM CO., LTD., China), ZF14 (n = 1.92, CDGM CO., LTD., China) and ZK9 (n = 1.62, CDGM CO., LTD., China) respectively for the prism 0, prism 1 and prism 2. The 3D model and a photo of the ASIL are shown in Fig. 3(e) and (f). Based on the optimization of above optical elements for the resonance condition of the PSPs, the incidence angle is achieved as about 32° in prism 0. Thus, the laser spot is calculated as about 4 μm in diameter (see Fig. S5). The long-pass filter (BLP01-785R-25, Semrock, Inc, USA) and the collection lens (f = 60 mm, K9, Daheng Optics, Inc, China) are installed in a lens holder which is fixed concentrically with the ASIL. The collection fiber (NA = 0.22, diameter = 100 μm, FIB-200-NIR, Ideaoptics, Inc, China) is installed on the fiber collimator to receive the SERS signal and transports the SERS to the Raman spectrometer (NA = 0.125, QE65000, Ocean Optics, Inc, USA). A tablet computer is integrated into the *i*PERS to control the system directly. The system is controlled by a LabVIEW program (National Instruments Co.) on the tablet computer. The casing of the *i*PERS is made up of aluminum. The NOF on the ZLaF4 disks were fixed on the top of Prism 0 with a refractive index liquid (methylene diiodide). The weight of the *i*PERS is only 4.5 Kg. And the whole dimension of the *i*PERS is 280 mm × 65 mm × 195 mm. It is a compact and portable analyzer for the SERS spectral measurement.

## Electronic supplementary material

supporting information
