# Supplementary material for: Integrated plasmon-enhanced Raman scattering (iPERS) spectroscopy
Source: Sci Rep. 2017 Nov 7;7:14630. doi: 10.1038/s41598-017-15111-3 (PMC5676962; doi:10.1038/s41598-017-15111-3)
Supplement: Supplementary file 1 — supporting information [file 41598_2017_15111_MOESM1_ESM.pdf]

Integrated Plasmon-enhanced Raman scattering (*i*PERS) spectroscopy

*Hailong Wang[1], Haibo Li[1], Shuping Xu[1], Bing Zhao[2], and Weiqing Xu[1]**

[1] State Key Laboratory of Supramolecular Structure and Materials, Institute of Theoretical Chemistry, Jilin University, Changchun 130012, People's Republic of China

[2] State Key Laboratory of Supramolecular Structure and Materials, College of Chemistry, Jilin University, Changchun 130012, People's Republic of China

S1: The vector-match law.

The coupling between the free propagation light and the propagating surface plasmon polaritons (PSPs) propagating along the noble metal film obeys the vector-match law. The vector-match law determines the excitation and emission ways of SERS signal from the Ag nanoparticle-on-a film (NOF) substrate, which means that the magnitude and orientation of the propagation wave vectors of the excitation and emission light equal that of the SPs, as shown in Fig.S1.[1-3] The vector-match law can be expressed as below. For the excitation process of PSPs on the upper surface of Ag film,

$$k_{psp1} = k_{in} \sin\theta \qquad (1)$$

and for the emission process of PSPs on the prism side,

$$k_{psp2} = k_{out} \sin\beta \qquad (2)$$

where θ and β are the excitation and emission angles of PSPs respectively. k_{psp1} and k_{psp2} are the propagation wave vectors of the PSPs on Ag surface with different

frequencies, which are expressed as

$$k_{Psp1} = \frac{\omega_1}{c}\sqrt{\frac{\varepsilon_m}{\varepsilon_m+1}} \tag{3}$$

$$k_{Psp2} = \frac{\omega_2}{c}\sqrt{\frac{\varepsilon_m}{\varepsilon_m+1}} \tag{4}$$

ω_1 and ω_2 are the different frequencies of PSPs. c is the light speed in vacuum. ε_m is the dielectric constant of silver, which is the function of frequency. The real part of the Ag dielectric function is shown as the black curve in Fig.S1b. For the integrated plasmon-enhanced Raman scattering (*i*PERS) instrument, the refraction index of the prism is 1.92. With equation (1) to (4), the excitation and directional emission angles for PSPs from the prism side are shown as the blue curve. The red region shows the main spectra acquired by the *i*PERS instrument, in which the excitation laser is 785 nm. The excitation and emission angles are below the red dash line (32.2 degree), which is covered by the large numerical aperture of the ASIL.

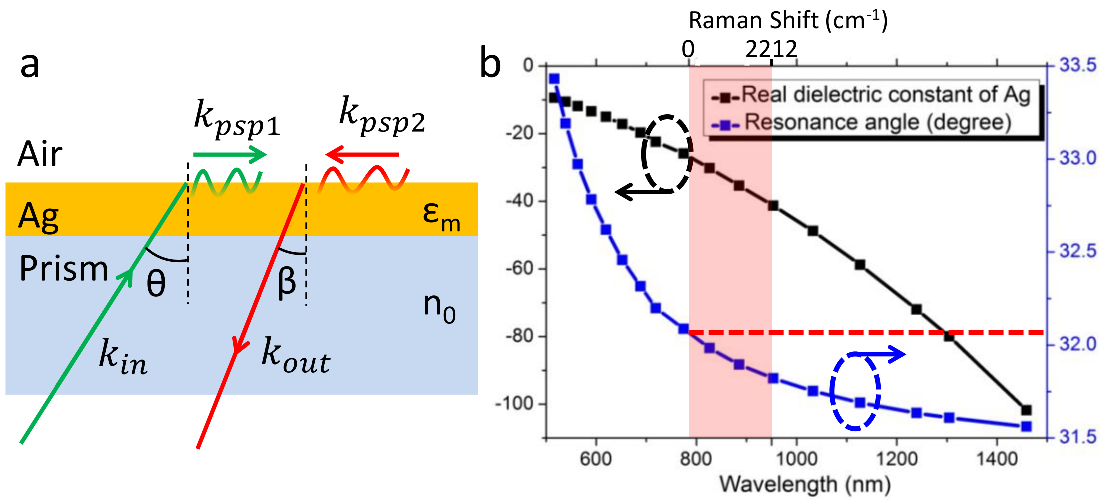

Figure S1. (a) The excitation and emission process of PSPs from the prism side. (b) The resonance angle of PSPs on the surface of the Ag film with different frequencies as the black curve. The dielectric constant data of the Ag is from Palik's book shown as the blue curve.[4]

S2: Directional emission of PSPs on the NOF structure.

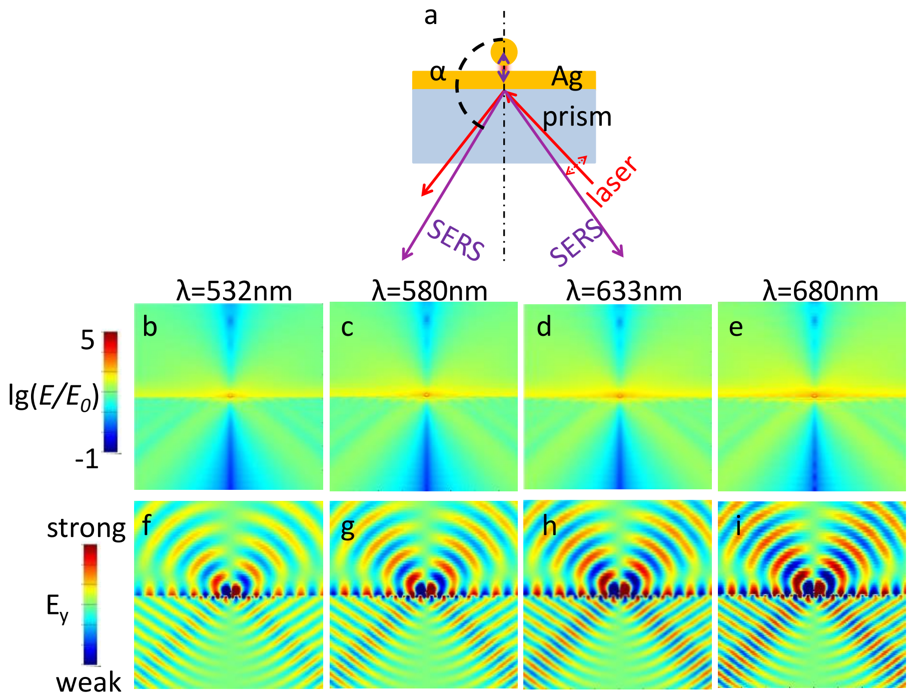

Figure S2. Directional emission patterns of different wavelength dipoles located in the gap of the NOF to mimic the emission process of the SERS photons. (a) Schematic diagram of the model for 2D-FDTD simulation. The dipole is located in the nanogap (1.0 nm), and α is the emission angle of the radiation ray (SERS in the figure). (b)-(e) The far field electric field distributions of dipoles with different wavelengths. The directional emission of SERS is also clearly shown in (f)-(i), while the component of electric field is parallel to the Ag film.

S3: The directional emission of PSPs on the Ag film substrate.

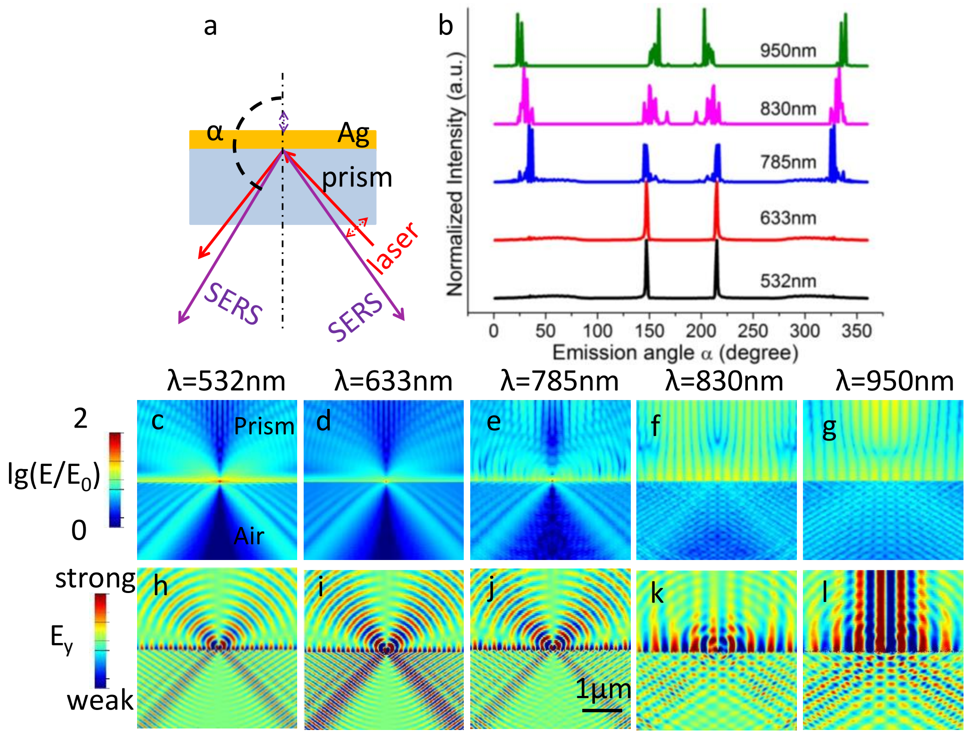

Figure S3. The directional emission patterns of different wavelength dipoles located over the Ag film to mimic the emission process of the SERS photons. (a) Schematic diagram of the model for 2D-FDTD simulation. The dipole is located at the surface of Ag film, and α is the emission angle of the radiation ray (SERS in the figure). (b) reveals the emission angles with different wavelength dipoles (532nm-950nm) in far field. Most of SERS radiates into a sharp cone angle in the prism side. (c)-(g) are the electric field distributions in far field of dipoles with different wavelengths. The directional emission of SERS is also clearly shown in (h)-(l), while the component of electric field is parallel to the Ag film.

S4: Basic properties of *i*PERS

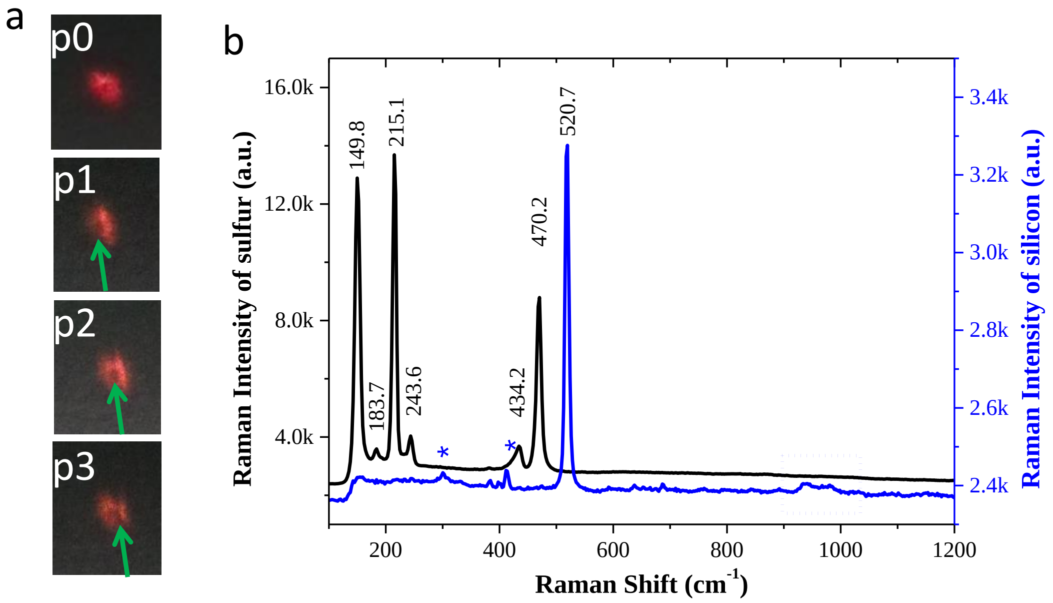

Figure S4. The SPR dark slit in the reflection light spots (a) shifts along with the incident angle increasing. p0 is the reflection light spot without the dark slit. p1, p2 and p3 are corresponding to the different incident angles. (b) is the Raman spectra of the sulfur crystal (dark line) and the silicon crystal (blue line) detected by the *i*PERS setup. The laser power is 11 mW and the integration time is 10s.

The basic properties are demonstrated in figure S4. As the *i*PERS setup is based on the SPR mechanism, the incident angle of the excitation laser need to be in the proper range. A smooth Ag film (thermal deposition; thickness: 40 nm) is utilized as the substrate, which is placed at the upper surface of prism 0. As shown in figure S4a, the dark slit due to the SPR is clearly visible in the reflection light spot (p1, p2 and p3) in contrast with the normal one (p0). The SPR band shifts from one side (p1) to the other side (p3) of the reflection light spot along with the incident angle increasing, which is consistent with the previous investigation by Pablo G. Etchegoin and

coworkers.[5] The dark slit of SPR clearly reveals that the *i*PERS supports the excitation of the PSPs on the smooth Ag film.

The collection performance of Raman spectra by the *i*PERS setup were checked (see figure S4b). The sulfur crystal and silicon single crystal are closely placed at the upper surface of prism 0. The space between the crystal samples and the prism 0 is filled with a matching liquid (arsenic tribromide and disulfide and sulfur, n=1.92). The spectrum of the sulfur crystal (black line) clearly shows the Eigen resonance at 149.8, 183.7, 215.1, 243.6, 434.2 and 470.2 cm^{-1}, which are coincident with the related results in the literature.[6] And the Raman shift of the monocrystalline silicon (blue line) at 520.7 cm^{-1} has the intensity of 891 counts per second. The second-order Raman shift (blue dot rectangle) of the monocrystalline silicon is clearly identified, which reveals the high collection performance of the *i*PERS in the basic mode.

S5. ASIL.

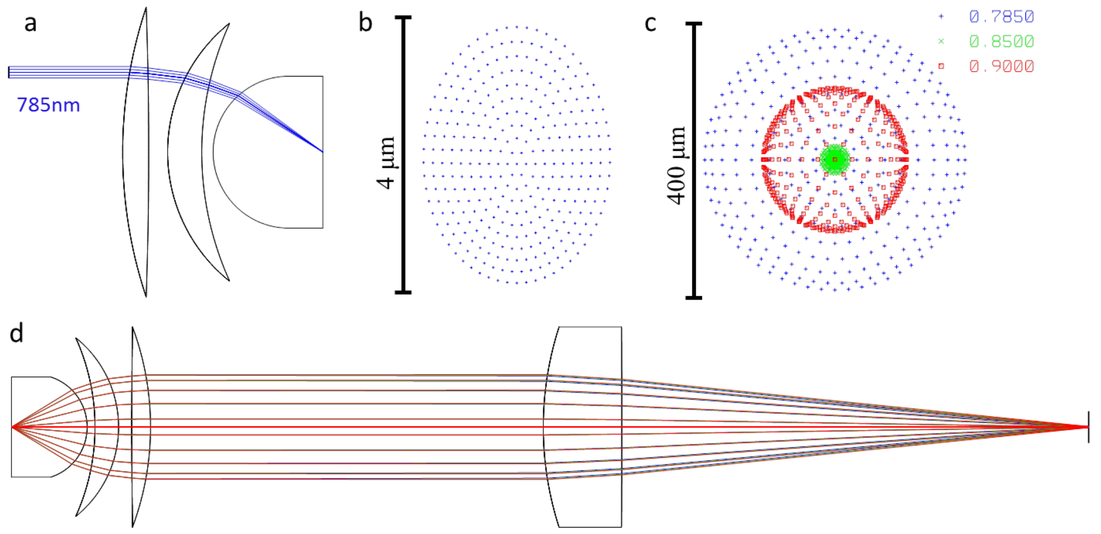

Figure S5. Two key procedure in the design of the ASIL and *i*PERS system. (a) The

incidence light path. (b) The calculated excitation laser spot. (c) The collection signal pattern after the collection lens focusing, which is a reference for the spectrometer collecting all the wavelength. Numbers with colors indicate wavelengths with a unit of μm. (d) The signal collection light path.

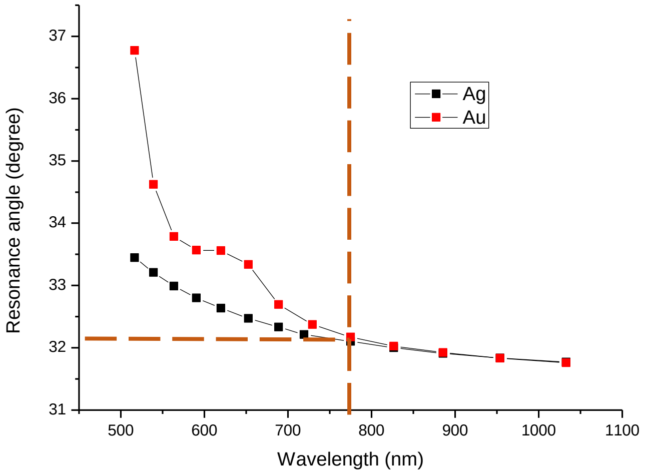

Figure S6. The calculated resonance angles for Ag and Au on *i*PERS.

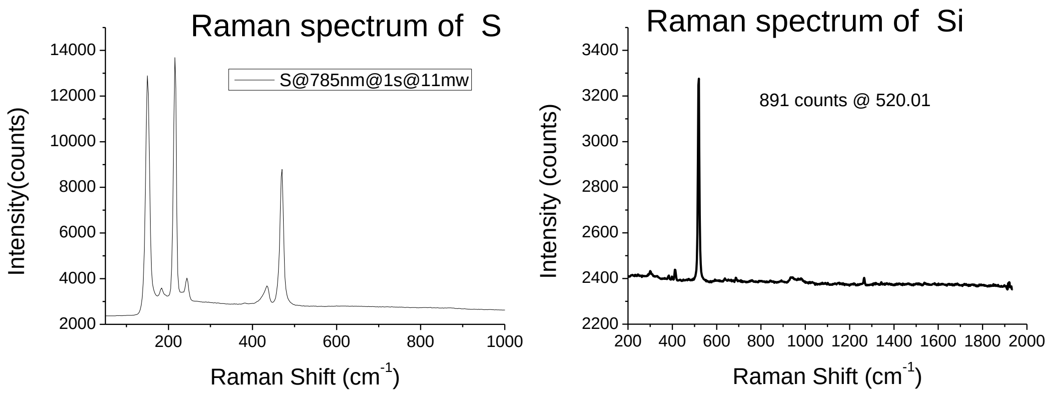

Figure S7. The Raman spectra of S and Si measured by the our designed ASIL lens and the *i*PERS collection system (without the NOF slide).

References:

1 Lakowicz, J. R. Radiative decay engineering 3. Surface plasmon-coupled directional emission. *Anal. Biochem.* 2004, **324**, 153-169.

2 Gryczynski, I., Malicka, J., Gryczynski, Z. & Lakowicz, J. R. Radiative decay engineering 4. Experimental studies of surface plasmon-coupled directional emission. *Anal. Biochem.* 2004, **324**, 170-182.

3 Cao, S. H., Cai, W. P., Liu, Q. & Li, Y. Q. Surface plasmon-coupled emission: what can directional fluorescence bring to the analytical sciences ? *Annu. Rev. Anal. Chem.* 2012, **5**, 317-336.

4 Palik, E. D. & Ghosh, G. *Handbook of optical constants of solids*. Academic Press, 1998.

5 Meyer, S. A., Le Ru, E. C. & Etchegoin, P. G. Combining surface plasmon resonance (SPR) spectroscopy with surface-enhanced Raman scattering (SERS). *Anal. Chem.* 2011, **83**, 2337-2344.

6 Ward, A. T. Raman spectroscopy of sulfur, sulfur-selenium, and sulfur-arsenic mixtures. *J. Phys. Chem.*1968, **72**, 4133-4139.
